# *DE*ep *VE*in *L*esion *OP*timisation (DEVELOP) trial: protocol for a randomised, assessor-blinded feasibility trial of iliac vein intervention for venous leg ulcers

**DOI:** 10.1186/s40814-021-00779-2

**Published:** 2021-02-04

**Authors:** Thomas M. Aherne, Colm Keohane, Matthew Mullins, Adeel S. Zafar, Stephen A. Black, Tjun Y. Tang, Gerard J. O’Sullivan, Stewart R. Walsh

**Affiliations:** 1 Lambe Institute for Translational Discipline of Surgery, National University of Ireland, Galway, Ireland; 2 Department of Vascular Surgery, University Hospital Galway, Newcastle Road, Galway, Ireland; 3 Department of Interventional Radiology, University Hospital Galway, Galway, Ireland; 4 Guy’s and St Thomas’ N.H.S. Foundation Trust, London, UK; 5 Department of Vascular Surgery, Singapore General Hospital, Singapore, Singapore

**Keywords:** Venous ulcer, Endovenous, Deep venous intervention, Iliac vein, Intra-vascular ultrasound, Saphenous incompetence

## Abstract

**Background:**

Venous leg ulceration is a widespread, debilitating pathology with high recurrence rates. Conservative treatment using graduated compression dressings may be associated with unacceptable ulcer recurrence rates. Early superficial venous ablation encourages ulcer healing and reduces recurrence. However, many of this cohort display concomitant ilio-caval stenosis, which further contributes to lower limb venous hypertension and ulceration.

An approach that combines early superficial venous ablation with early treatment of ilio-caval stenotic disease may significantly improve ulcer healing and recurrence rates. We question whether early iliac vein interrogation with intravascular ultrasound (IVUS), stenting of significant occlusive disease plus superficial venous ablation, in patients with active venous leg ulceration, will produce superior ulcer healing to standard therapy.

**Methods:**

This is a prospective, multi-centre, randomised controlled, feasibility trial recruiting patients with lower limb venous ulceration and saphenous venous incompetence. Patients will be randomised to undergo either truncal ablation and compression therapy or truncal ablation, simultaneous iliac interrogation with intravascular ultrasound and stenting of significant (> 50%) iliac vein lesions plus compression therapy. The primary feasibility outcome will be the rate of eligible patient participation while the primary clinical outcomes will be ulcer healing and procedural safety. Secondary outcomes include time to healing, quality of life and clinical scores, ulcer recurrence rates and rates of post-thrombotic syndrome. Follow-up will be over a 5-year period. This feasibility trial is designed to include 60 patients. Should it be practicable a total of 594 patients would be required to adequately power the trial to definitively address ulcer-healing rates.

**Discussion:**

This trial will be the first randomised trial to examine the role iliac interrogation and intervention in conjunction with standard operative therapy in the management of venous ulceration related to superficial truncal venous incompetence.

**Ethical committee reference:**

C.A. 2111 Galway Clinical Research Ethics Committee

**Registration:**

Clinical Trials.gov registration NCT03640689, Registered on 21 August 2018.

**Supplementary Information:**

The online version contains supplementary material available at 10.1186/s40814-021-00779-2.

## Background

Leg ulcers are a widespread, debilitating problem with a prevalence in Ireland of 0.12% overall. This increases to 1% among patients over 70 years of age. Recurrence is common with 12-month healing rates reported between 16 and 36% [1]. The majority of these leg ulcers (81%) are venous in origin and are treated in the community by public health nurses at a significant annual cost [2].

Typically chronic venous hypertension results in lower limb skin changes, often at the level of the ankle. Oedema, fibrin pericapillary cuffs and/or trapping of white cells within the interstitium typically precede tissue loss with these changes leading to skin ulceration as a result of relative local tissue hypoxia or cytokine/protease release [3, 4].

The mainstay of treatment of leg ulcers is the application of graduated compression to the limb with the aim of promoting venous return, reversing venous hypertension and the local tissue changes, thus allowing the ulcer to heal. Maximum compression is applied at the ankle with gradually lesser compression being applied proximally up the leg. Graduated compression bandaging is usually used to achieve ulcer healing while compression stockings are fitted to prevent recurrence. A Cochrane review of the management of leg ulcers concluded that compression increases ulcer healing rates compared to no compression, multi-layered systems are more effective than single-layered systems and that high compression was better than low compression [5]. Nevertheless, in spite of the application of best evidence-based therapy, healing rates for venous leg ulcers remain disappointing at 50% to 70% following 12 weeks of treatment [6].

With an increasingly elderly population, the incidence of venous ulceration is likely to rise. The negative impact of leg ulceration on patients’ quality of life and on healthcare costs is well recognised [7–9]. Increased prevalence combined with poor reported healing rates and high incidence of ulcer recurrence makes the development of a new treatment which could accelerate healing rates beyond that currently achieved using compression bandaging most desirable.

## Trial rationale

Traditionally, venous reflux was viewed as the primary causative factor in venous ulceration. Treatment strategies such as compression or, more recently, early superficial venous ablation [10] aim to address the reflux and thereby encourage ulcer healing. However, advances in imaging techniques have revealed that ilio-caval venous obstruction occurs frequently in patients with chronic venous disease [11, 12]. Moreover, endovascular treatment of ilio-caval occlusive disease produces significant symptomatic improvement even in the presence of persistent uncorrected deep venous reflux [13].

Two recent systematic reviews have concluded that endovascular stent placement for iliac-caval venous obstruction is safe with high technical success rates [14, 15]. Non-thrombotic iliac vein lesions (NIVLs) are present in up to 80% of patients with symptomatic venous disease [16]. These lesions are characterised by their non-thrombotic aetiology and often are identified at post-mortem as intravascular adhesions or membranes. Commonly, they are in the form of a web and may progress to venous occlusion or contribute to increased lower limb venous pressures. These obstructive lesions, along with post thrombotic stenoses, are left unaddressed by current ulcer treatment strategies, which focus solely on the reflux component of the underlying venous hypertension. This predisposes to persistent venous hypertension, in turn leading to recurrent superficial venous reflux and an increased risk of recurrent ulceration. This focus solely on the reflux component may explain the relatively limited improvement in ulcer healing rates in studies of early venous ablation [10].

An approach which combines early superficial venous ablation with early treatment of iliac occlusive disease, i.e. addresses both the reflux and obstructive components of the disease, could significantly improve ulcer healing and recurrence rates. Iliac vein interrogation and stenting if appropriate undertaken simultaneously with superficial venous ablation has the potential to significantly improve ulcer healing rates.

Thus, we ask, that in patients with active venous leg ulceration does early iliac vein interrogation with intravascular ultrasound (IVUS), stenting of significant occlusive disease plus superficial venous ablation produce superior ulcer healing to compression therapy plus superficial venous ablation alone?

## Objectives

The single main research question for this feasibility trial is as follows: in adult patients with venous ulceration and saphenous vein incompetence is iliac vein assessment with IVUS and stenting (of significant occlusive disease) in addition to saphenous venous ablation and compression feasible and acceptable to patients with venous ulceration?

### Primary objective

The primary obective is to determine the proportion of suitable patients with venous ulceration who agree to randomisation to either superficial venous ablation plus early iliac vein interrogation plus endovascular stenting in the presence of significant occlusive disease or to superficial venous ablation plus compression therapy alone.

### Secondary objectives

The secondary objectives are the following:

1. To determine the relative performance of duplex ultrasound compared to IVUS for the prediction of occult iliac disease
2. To determine the rate of primary or recurrent ulceration up to 5 years following intervention
3. To assess patient quality of life in the short and medium term following each mode of intervention
4. To determine what proportion of patients require iliac vein stenting on the basis of IVUS
5. To determine the outcomes of routine IVUS in patients with venous ulceration
6. To determine the safety of routine IVUS and/or iliac vein stenting

## Trial design and endpoints

### Statement of design

This is a prospective randomised controlled, assessor-blinded, feasibility trial with participants allocated to one of two parallel groups in a 1:1 fashion. The primary trial centres will be the University Hospital Galway, Ireland, and Singapore General Hospital with further centres currently being evaluated for inclusion. Both the Departments of Vascular Surgery and Interventional Radiology will conduct the trial in unison with equal oversight. Suitable patients shall be recruited from both the outpatient and inpatient setting.

### Outcomes

#### Primary feasibility outcome

The primary feasibility outcome of this study will be the proportion of eligible patients with venous ulceration who agree to proceed with intervention and undergo randomisation. The proportion of eligible patients for inclusion will also be recorded to inform the rate of potential recruitment. An acceptance rate of 50% among those eligible will be deemed feasible.

#### Primary efficacy outcomes

The primary efficacy outcome will be the proportion of ulcers healed 3 months following the overall intervention and the iliac vein patency in the interventional group.

- A healed ulcer is defined as complete re-epithelialization with no scab and no requirement for dressing [17].

Stent patency will be evaluated by ultrasound within 24 h, at 6 months, 1 year and annually thereafter. One-year patency rates will be reported alongside the primary efficacy endpoint. Five-year patency rates will be reported in a separate long-term follow-up report. The following will be reported with respect to patency:

- Primary patency: patent stent with less than 20% in-stent or between stent stenosis and no re-interventions
- Primary assisted patency: patent stent with less than 20% in-stent or between stent restenosis which has undergone re-intervention to prevent occlusion
- Secondary patency: patent stent which has been reopened following occlusion
- Stent occlusion: stent with a no-flow segment which has not undergone re-intervention or in which re-intervention failed

#### Primary safety outcomes

The primary safety endpoints in the intervention group are any complications arising from the additional use of IVUS and the requirement for subsequent acute or sub-acute re-intervention (at any time) as a result of the primary intervention.

##### Re-intervention

Freedom from re-intervention will be presented for 1 -year and 5-year follow-up reports. Both superficial and deep venous re-interventions will be recorded.

#### Secondary outcomes

1. Percentage reduction in ulcer area at 12 weeks, 6 months and 1 year
2. Time to ulcer healing (days)
3. Bates-Jensen wound assessment tool (BWAT) [18, 19] scores for the index ulcer at 6 weeks, 12 weeks and 6 months
4. Changes in Venous Clinical Severity Score [20] at 6 weeks, 3 and 6 months and 1 year.
5. Number of healthcare contacts in the 6 months following the procedure
6. Changes in EQ5D [21] measures of quality of life at 6 weeks, 12 weeks, 6 months and 1 year
7. Ulcer recurrence following healing of the index ulcer
8. Unplanned hospital admission in 6 months following the procedure
9. Health economic analysis based on generated data
10. The number of patients who require additional iliac stenting as a result of additional IVUS
11. The rate at which patients complete their allocated intervention
12. The numbers of patients who are compliant with assigned follow-up and wound dressing regimes.
13. The identification of challenges to patient recruitment and follow up compliance

## Trial population

### Inclusion criteria

Consenting patients, aged 18 and over, with ultrasound detected great or short saphenous venous incompetence with an associated primary or recurrent lower limb venous ulcer(s) will be eligible for inclusion. Saphenous reflux is defined as retrograde flow > 0.5 s in superficial vein [17].

### Exclusion criteria

1. Ankle-brachial pressure index < 0.8
2. Previous inability to tolerate lower limb compression bandaging
3. Inability to provide informed consent
4. Previous lower limb arterial revascularisation procedure
5. Contrast allergy
6. Previous history of pelvic malignancy or pelvic radiotherapy
7. Current pregnancy
8. Previous iliac vein intervention
9. Previous Saphenous vein intervention
10. Infection in the previous 30 days
11. Estimated glomerular filtration rate (eGFR) < 60 ml/kg/min
12. Perforator vein reflux
13. Leg ulcer of non-venous aetiology (as assessed by clinician)
14. Unfit for endovascular intervention based on history and examination
15. Contraindications to anticoagulation

### Informed consent of the inpatient

The process of obtaining informed consent will be conducted in compliance with the principals of good clinical practice and requirements of the approving research ethics committee. Consent to enter the trial will be sought from each subject only after a full written and verbal explanation has been given and appropriate time allowed for consideration. The consent process will be conducted by the trial investigators in all cases. Signed subject consent will be obtained and stored in the medical notes. It is a right of the subject to refuse to participate or to withdraw at any time from the protocol without giving reasons and without prejudicing treatment.

#### Informed consent of the outpatient

Eligible patients will be given an information sheet at their outpatient visit and the trial will be explained to them by a team member. An anaesthetic pre-assessment visit will be conducted if applicable. Written informed consent will be obtained immediately before the procedure by an investigator.

### Randomisation

Following the consent process randomisation will be undertaken using sequentially numbered opaque sealed envelopes administered by an independent research assistant in the Lambe Institute, University Hospital Galway, allowing for allocation concealment. A unique trial number will be assigned to each individual at the time of randomisation. Patients will be randomised to one of two groups.

Group 1: Compression bandaging and endovascular treatment of saphenous truncal incompetence.

Group 2: Compression bandaging, endovascular treatment of saphenous incompetence with simultaneous iliac vein interrogation with IVUS and stenting if an occlusive lesion > 50% is identified.

A designated member of the research team who will not be involved with data collection will have access to the randomisation data and arrange intervention and investigation as required. There will be no sham intervention.

### Baseline patient data

Patients will all have a full medical history taken and clinical examination as part of their standard care. The following will be recorded:

1. Weight
2. Height
3. Blood pressure
4. Heart rate
5. ECG
6. Gender
7. Ethnicity
8. Date of birth
9. Diabetes mellitus
10. Hypercholesterolaemia
11. Hypertension
12. Previous myocardial infarction
13. Previous coronary revascularisation
14. Previous stroke
15. Atrial fibrillation
16. Peripheral arterial disease
17. Smoking history
18. NYHA class
19. Bleeding history
20. History of deep venous thrombotic events
21. Obstetric history
22. Mobility (scored 1–4; 1, full; 2, moderate—able to perform most everyday tasks with some impairment; 3, severe—can only mobilise very short distances such as bed to chair; 4, no mobility)
23. Medication at time of consent (aspirin, clopidogrel, beta-blocker, calcium-channel antagonist, nitrates, cholesterol-lowering agent, ACE inhibitor/A2 receptor antagonist, insulin, metformin, sulphonylurea, warfarin)
24. Duration of ulcer prior to enrolment in trial
25. Venous Clinical Severity Score/EQ5D

#### Ulcer assessment

**Fig. 1:**
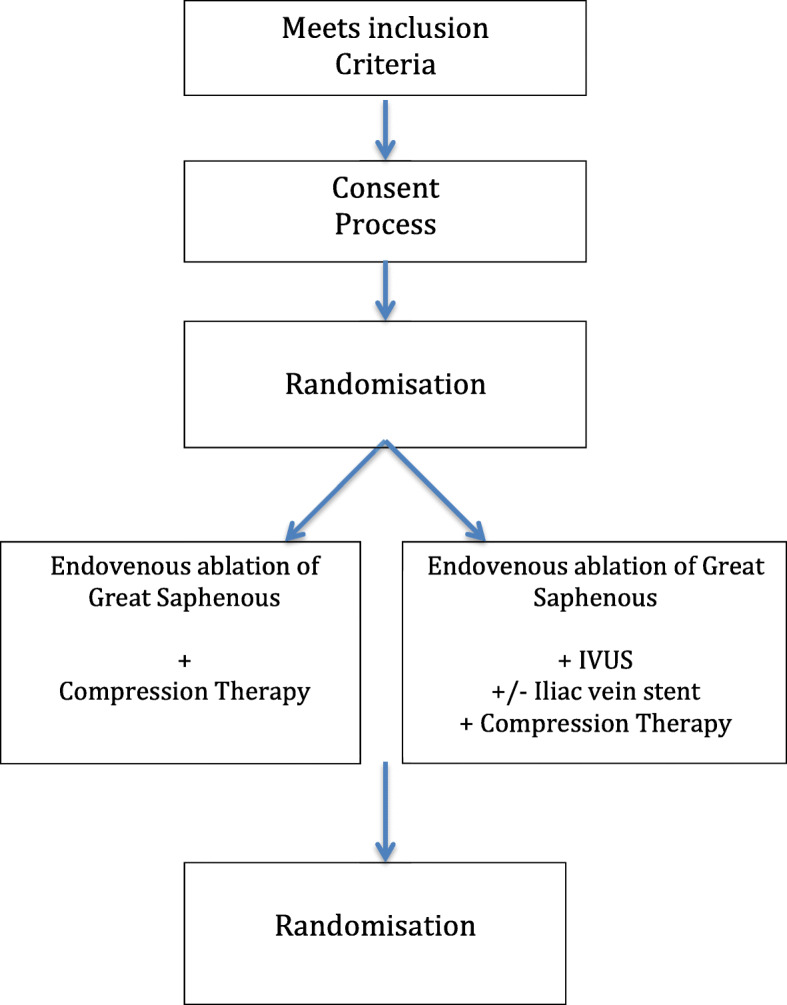
Schematic of study flow

The ulcer will be evaluated by an assessor who has been blinded to the treatment allocation of the patient. Wound size will be calculated using digital planimetry and recorded as cm^2^. Wound assessment will be evaluated using the Bates-Jensen wound assessment tool which has been validated for use in venous leg ulcer assessment [18, 19]. Ulcers that heal prior to intervention will be analysed with intention to treat (Fig. 1).

#### Ankle-brachial pressure index

All patients in whom pedal pulses are impalpable will have an ankle-brachial pressure index calculated using a handheld Doppler and a manual sphygmomanometer.

#### Duplex ultrasonography

As part of routine clinical care, all patients with a venous leg ulcer undergo a lower limb venous duplex ultrasound. This will assess both the deep and superficial venous systems of the affected leg. The results from this scan will be used as the baseline pre-treatment lower limb duplex result.

#### Feasibility data

1. The number of eligible patients
2. The number of consenting patients
3. The number of patients declining participation
4. Assessment of the number of patients who attend or miss scheduled follow-up
5. Reasons for missed follow-up appointments
6. Barriers to timely scheduling of interventions and follow-up

## Interventions

### Endovenous ablation and IVUS procedure

All procedures will be carried out by either a vascular surgeon or interventional radiologist with significant experience in both endovenous ablation and IVUS (Visions 0.035, Philips, Amsterdam). The aim is for ablation within 4 weeks of randomisation. All patients will undergo endovenous ablation of refluxing axial superficial veins in the affected leg using thermal or non-thermal ablative techniques under local anaesthesia. Modality of intervention will be at the discretion of the operator. Control procedures will be undertaken in an ambulatory care setting while the interventional group will be managed in an operating theatre or interventional suite. All procedures will be completed under local anaesthesia.

The ablation procedure will be performed simultaneously with IVUS and stenting where applicable. A 9F access sheath will be placed under ultrasound guidance in the saphenous vein at the lowest point of reflux to allow introduction of firstly the IVUS catheter into the ipsilateral common iliac, external iliac and common femoral veins followed by the ablation probe. If the IVUS cannot be passed, the common femoral or jugular vein will be cannulated at the discretion of the operator. Iliac vein interrogation using the IVUS will be undertaken to determine the degree of area luminal area reduction (LAR) at each of the known arterial and ligamentous crossing points on the affected side which predispose to NIVL formation. These include the left and right proximal common iliac veins (where crossed by the right iliac artery), the right and left proximal external iliac vein (where crossed by the external iliac arteries and the retro-inguinal regions.

The LAR will be determined by comparison of the minimal luminal area at the NIVL site to the anatomical minimum for the respective vein, as recommended by experts in the field [22]. Area will be calculated using the standard mathematical formula Area = *A* = *πr*^2^. The reference area values for the iliac veins and the common femoral veins are detailed in Table 1. Lesions will be classified into the degree of stenosis by luminal area as detailed in Table 2.

**Table 1:** Normal anatomical reference values for deep veins

Vein	Normal minimum diameter	Normal minimum area
**Common iliac vein**	16 mm	201 mm^2^
**External iliac vein**	14 mm	154 mm^2^
**Common femoral vein**	12 mm	113 cm^2^

**Table 2:** Degree of stenosis based on luminal area

Vein	< 20% stenosis	20 to 49% stenosis	50 to 69% stenosis	> 70% stenosis
**Common iliac vein**	≥ 161mm^2^	160 to 103 mm^2^	102 to 62 mm^2^	≤ 61 mm^2^
**External iliac vein**	≥123mm^2^	122 to 79 mm^2^	78 to 48 mm^2^	≤ 47 mm^2^
**Common femoral vein**	≥ 90 mm^2^	89 to 58 mm^2^	57 to 35 mm^2^	≤ 34 mm^2^

Patients with a stenosis < 50% on the affected side will not have any further iliac or common femoral vein procedure performed. They will undergo endovenous ablation via the previously placed sheath in the great saphenous vein at the knee. Patients with a lesion > 50% will undergo iliac vein stenting with subsequent great saphenous ablation. The venous stent utilised will be at the discretion of the operator to broaden applicability. All residual varicosities shall be treated with foam sclerotherapy using 1% polidocanol. Foam will be standardised with 1:4 polidocanol to air ratio with 2 ml’s administered to each varicosity. Post-operatively those undergoing venous stenting will be placed on a three-month course of a novel oral anticoagulant.

## Compression

### Compression therapy

All groups will have four- or two-layer compression (Profore, Smith and Nephew, UK/Coban, 3M, USA) bandaging applied upon initial review once arterial supply is deemed adequate. All patients will be placed in two-layer bandaging immediately post-procedure which will be replaced with a four-layer bandage within 48 h. Specialist nurses in wound management will subsequently manage compression bandaging in the community with regular review by a vascular surgeon.

## Schedule of events

### Follow-up

All patients will be fully assessed at weeks 6 and 12. Further assessments will be undertaken at 6 and 12 months and annually thereafter. Follow-up will be carried out by assessors blinded to the initial intervention.

### Ulcer assessment

Baseline assessment will be conducted as described in the ‘Randomisation’ section.

The following end-points will be evaluated at each visit:

- Integral of the relative ulcer size (area) for each patient over time, standardised to an initial size of 1
- Duration until complete wound closure
- Assessment of wound edges Assessment of granulation (BWAT)
- Assessment of exudation (BWAT)
- Assessment of wound edges (BWAT)
- Assessment of undermining (BWAT)
- Assessment of wound bed tissue (BWAT)
- Maximum pain in 24 h prior to each visit (0 to 100 VAS)

### Venous clinical severity score

The Venous Clinical Severity Score will be completed at 6 and 12 weeks initially with further assessment at 6- months and annually thereafter until 5-year follow-up is complete.

### EQ5D questionnaire

Quality-of-life will be evaluated by completion of the EQ5D form at 6 weeks and 12 weeks, 6 months and annually thereafter until 5-year follow-up is complete. This data will be further utilised to generate health economic data.

### Healthcare contacts diary

Each participant will be provided with a diary following their venous ablation procedure in order to record all planned and unplanned healthcare contacts (GP visits, public health nurse visits, emergency department visits, hospital admissions, outpatient visits) and ulcer healing data. Community nursing will also report real-time ulcer healing centrally prompting clinical review. The patients will be asked to fill out the diary until their 6-month visit.

### Iliac stent follow-up

Patients who undergo iliac vein stenting alone will undergo follow up iliac vein colour duplex in the radiology department at 1 day, 6 months, 1 year and annually thereafter. Reflux duration, stent patency, stent fracture, in-stent or between stent restenosis will be recorded at each visit. Due to the nature of this surveillance, it will not be possible to blind the ultrasonographer who will not be involved in the trial protocol.

### Superficial venous ablation surveillance

All patients undergoing endovenous ablation will undergo duplex ultrasound assessment of the treated great saphenous vein at 6 weeks and 6 months to assess for recanalisation.

### Crossovers

Any crossovers between treatment groups shall be highlighted and reported in the final report. All patients in the control group with unhealed ulcers will be offered further iliac investigation and treatment where necessary at 6 months. Furthermore, control patients with a history of iliofemoral DVT will be offered investigaton and/or treatment at 3 months due to their higher risk of occlusive disease.

### Withdrawals during follow-up

An individual is free at anytime to withdraw from the trial for any reason without any prejudice as to subsequent therapy. The principal investigator may withdraw and individual from the trial should they be subject to an adverse event whereby it is in the patients best interest to be withdrawn or should they meet any of the exclusion criteria.

### Loss to follow-up

Prior to consent participants will be educated as to the importance and timing of the follow-up protocol. Post-intervention regular patient contact in the community through specialist nurses will allow for a continued patient dialogue regarding follow-up. Should follow-up appointments be missed investigators will endeavour to contact patients by phone or via community liaisons to ensure timely follow-up within the trial protocol. All losses to follow-up and its cause shall be recorded and reported in any trial data.

### Protocol violations

The following will be deemed a violation of protocol and result in removal from the trial

1. Non-compliance with compression therapy despite adequate patient education
2. Failure to undergo ablation therapy within 6 weeks

### Premature termination of the study

The trial may be temporarily suspended or prematurely terminated if there is sufficient reasonable cause. Written notification, documenting the reason for trial suspension or termination will be provided to the regional ethical committee and all trial investigators by the principal investigator.

Circumstances that may warrant termination or suspension include, but are not limited to:

- Determination of unexpected, significant, or unacceptable risk to participants
- Demonstration of efficacy that would warrant stopping
- Insufficient compliance to protocol requirements
- Data that are not sufficiently complete and/or evaluable
- Determination of futility

### Schedule of events

The Appendix shows the schedule of events.

## Safety parameters

### Potential adverse events related to intervention

Primary safety endpoints include the need for re-intervention and stent patency. The risk of any significant adverse event occurring is deemed unlikely however all reasonable measures to avoid these events will be undertaken. Any adverse events (as described below) will be recorded and reported in any trial data.

1. Pain
2. Bleeding
3. Infection
4. Phlebitis
5. Nerve injury (temporary/permanent)
6. Incomplete ablation of targeted superficial vein
7. Recurrence of ulceration
8. Deep venous thrombosis
9. Pulmonary embolism
10. Adverse reaction to local anaesthetic
11. Iliac vein injury
12. Failure of stenting procedure
13. Pressure ulceration from dressings
14. Contrast-induced renal injury
15. Stent occlusion
16. Bleeding associated with anticoagulation

### Definition of an adverse event

The definition of an adverse event is any untoward medical event related directly or indirectly to an intervention as a result of participation in the trial.

### Definition of a serious adverse event

Any event that results in death, a life-threatening adverse event, inpatient hospitalisation or prolongation of existing hospitalisation, a persistent or significant incapacity or substantial disruption of the ability to conduct normal life functions, or a congenital anomaly/birth defect is deemed a serious adverse event.

### Event reporting

All adverse events will be reported directly to either the site or principal investigator. Recurrent adverse events or serious adverse events in isolation will be reported in turn to the local ethical committee and the risk assessment department for risk analysis. Indications for premature trial cessation are discussed in the ‘Protocol violations’ section.

### Trial audit

Regular audit of trial conduct shall be undertaken throughout the study period. The project management group (TA, CK, GOS, SRW) shall convene monthly to review trial proceedings internally and address any methodological, clinical and ethical concerns. External review shall be undertaken every 6 months by the local research ethics committee with further data monitoring from an independent research advisor from the National University of Ireland, Galway.

## Statistical considerations

Once randomisation and intervention is complete, patient outcomes will be assessed using an intention-to-treat analysis. All losses to follow-up at any time-point will be recorded and reported. A research statistician will conduct all analyses independently. Statistical analysis will be conducted using SPSS Version 21 (IBM Corp, Aramonk, NY) The primary feasibility outcome will be reported as the percentage of all eligible patients who agree to participate in the feasibility study. Descriptive data will be provided at baseline and at each follow-up to define variations among and within groups. Continuous data will be presented as mean and standard deviations with confidence intervals of 95%. Categorical data will be reported as frequencies with chi-square or Fischer’s exact *t* test used for comparative data. Where data is not normally distributed, non-parametric tests will be utilised as applicable. Further subgroup and regression analyses will be undertaken to adjust for confounding variables known to affect ulcer healing including age, duration, mobility and size. A *p* value of < 0.05 will be deemed significant.

Contemporary data suggests a median healing time of about 20 weeks for patients with a venous ulcer treated by superficial venous ablation [23]. Assuming a median time to healing in the control arm (those treated with superficial venous ablation and compression alone) of 140 days would be reduced to 110 days by the addition of iliac vein intervention where necessary (i.e. a 20% relative reduction in healing time) would require 270 patients in each arm of the trial, i.e. 540 in total. Allowing for a 10% withdrawal and dropout rate increases the required sample size to 594 patients. In advance of undertaking such a large trial, we aim to undertake a feasibility phase in order to assess eligibility, recruitment rates and provide baseline data for a refined sample size calculation. For this feasibility phase, we aim to recruit 30 patients to each trial arm.

### Interim analysis

Once 30 patients have been recruited and followed up for 6 months an interim analysis shall be undertaken to guide recruitment and further inform trial design with a view to powering the trial appropriately.

## Ethical considerations

### Ethical approval

Ethical approval has been approved centrally by the Galway Clinical Research Ethics Committee, Galway, Ireland. The ethics committee reference is C.A. 2111. Of note, recruitment shall not commence at any other centre until full ethical approval has been confirmed with the local ethical committee.

### Data protection

All data shall be managed in the strictest confidence by approved trial investigators in accordance with Irish data-protection law. Datasets will be anonymous, encrypted and stored onsite only.

## Discussion

The management of lower limb venous ulceration continues to raise significant debate particularly, in more recent years, with regard to adjunctive surgical intervention. At present, conservative measures including regular ulcer dressing with compression bandaging are well established. This approach offers undoubted benefits improving both ulcer healing rates and reducing recurrence [5, 24] in the longer term. However, this approach can be slow with a median ulcer healing time of up to 99 days [5] and high rates of non-compliance [24]. As such, more efficient treatment protocols continue to be investigated.

The recent publication of the EVRA trial [17] has further enhanced the concept that early surgical intervention can improve treatment protocols in the venous ulcer cohort. Included patients randomised to the intervention group of early endovenous intervention in conjunction with compression therapy experienced a significantly shorter time to ulcer healing. The authors identified a median ulcer healing time of 56 days in the treatment group compared to 82 in the control group among 450 patients. Furthermore, they identified an impressive overall healing rate of 85.6% at 24 weeks compared to 76.3%. This data consolidates evidence from the ESCHAR study [25] which confirmed lower ulcer recurrence rates at 12 months following combined intervention with surgery and compression therapy. As a result of these data a combined approach to venous ulceration is now widely advocated [26].

Thus, while it would appear that dual intervention offers benefit to ulcer patients a significant proportion of those affected may have concomitant NIVL’s [16] contributing to the venous hypertensive pathophysiology. This further exposes patients to delayed, non-healing and recurrent ulceration. At present, these lesions go largely unnoticed due to inadequate peri-operative imaging with access to MRV and CTV often resulting in further treatment delays and the added risks of contrast exposure. Intravascular ultrasound offers proven [27], minimally invasive imaging of the ilio-femoral segment simultaneously with superficial venous ablation. This provides the treating physician with a real-time assessment of NIVL’s and the ability to concomitantly treat significant lesions thus reducing risk of persistent venous hypertension.

This randomised controlled feasibility trial aims to provisionally assess the merits and safety of adjunctive procedural IVUS, stenting, endovenous ablation and compression therapy in the treatment of venous ulceration. To date, there is a paucity of data addressing this important health issue, and consequently, there is a need for well-designed large randomised studies examining the hypothesis.

Due to the significant number of patients required to adequately power this trial an initial feasibility trial on 60 patients will be carried out. This period will be used to assess trial flow, recruitment and the initial efficacy of treatment. Should the initial trial be successful the authors plan a multi-centre approach to allow for adequate and timely patient recruitment and treatment with a view to definitively identifying the role of iliac intervention in this cohort.

## Status of trial

Protocol: 1. V8

Recruitment: Commencing July 2019

Estimated Completion: September 2020

### Supplementary Information

**Additional file 1.** Research consent and information form.

## Figures and Tables

**Table 3:**
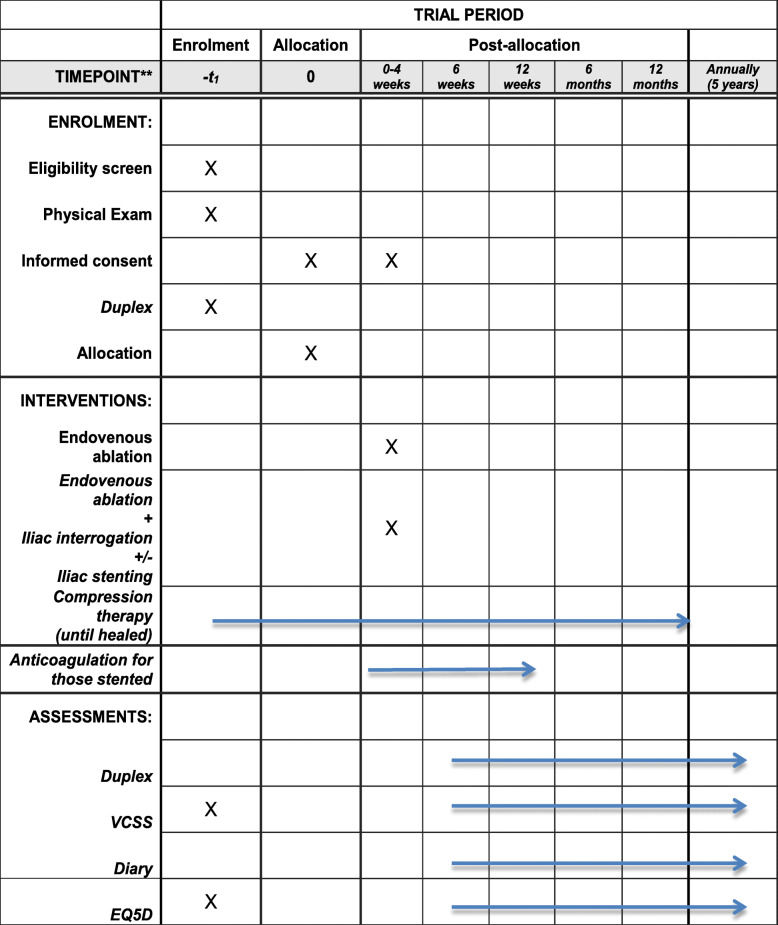
SPIRIT table

## Data Availability

Anonymous data and materials will be encrypted and stored in the secure server within the Lambe Research Institute, National University of Ireland. Data will be available upon suitable request to the senior author.

## Abbreviations

ABPI: Ankle-brachial pressure index
CT: Computed tomography
eGFR: Estimated glomerular filtration rate
LAR: Luminal area reduction
MR: Magnetic resonance
NIVL: Non-thrombotic iliac vein lesion
PTS: Post-thrombotic syndrome
BWAT: Bates-Jensen wound assessment tool
IVUS: Intravascular ultrasound
