# Supplementary material for: DEep VEin Lesion OPtimisation (DEVELOP) trial: protocol for a randomised, assessor-blinded feasibility trial of iliac vein intervention for venous leg ulcers
Source: Pilot Feasibility Stud. 2021 Feb 4;7:42. doi: 10.1186/s40814-021-00779-2 (PMC7860223; doi:10.1186/s40814-021-00779-2)
Supplement: Supplementary file 1 — Additional file 1. Research consent and information form. [file 40814_2021_779_MOESM1_ESM.docx]

# Research Consent and Information Form

**Title of Research Project:**

DEep VEin Lesion OPtimisation (DEVELOP) Trial: Study protocol for a randomised, assessor-blinded feasibility study of iliac vein intervention for venous leg ulcers

**Investigator(s):**

**Principal Investigator:**

- Dr Thomas Aherne, Specialist Registrar in Vascular Surgery, University Hospital Galway, Newcastle Road, Galway.

**Clinical Supervisor/Trial Lead:**

- Professor S Walsh, Consultant Vascular Surgeon, Department of Vascular Surgery, University Hospital Galway, Newcastle Road, Galway.

**Purpose of the Research:**

- Venous leg ulcers are a widespread, debilitating problem. They affect 1% among patients over 70 years of age. Recurrence is very common and attaining ulcer healing is challenging
- An approach to venous ulcers which combines early superficial venous ablation (keyhole surgery for varicose veins) with immediate treatment of iliac stenosis (narrowing in pelvic veins) addresses both the reflux and obstructive components of the disease which may significantly improve ulcer healing and recurrence rates. Thus examining the iliac vein with ultrasound and stenting narrowings in conjunction with superficial venous ablation has the potential to significantly improve ulcer healing rates.
- We hypothesise that in patients with active venous leg ulceration, early iliac vein interrogation with intravascular ultrasound and stenting of significant occlusive disease plus superficial venous ablation will produce superior ulcer healing to compression therapy plus superficial venous ablation.

**Description of the Research:**

• In this research study all patients with a venous ulcer undergoing minimally invasive treatment for varicose veins will be asked to participate. This process will commence in the outpatient department whereby the study and its significance will be explained to you. Should you have any queries or concerns these will be addressed at this point. Upon leaving the clinic you will be given written information about the trial to discuss further with others if required.

- On the day you present for your surgery the admitting physician will verbally re-consent you for participation in the study. If you have any further question they will be answered at this time. **You are free to withdraw your consent at any stage** and this will have no effect on your treatment schedule or the procedure itself.
- Once you confirm your participation you will be “randomized” to a treatment or control group. This means that you will be randomly placed in either a study group (where you will have minimally invasive treatment of your veins with an examination of your iliac vein using an ultrasound and stenting of any significant narrowings to relieve venous pressure in your leg) or a control group (where you will have minimally invasive treatment of your varicose veins). The “randomization” process is computer generated and those following you up will not know which group you are in.
- Prior to the procedure some basic information including your age, medical history and venous anatomy will be documented anonymously.
- Your procedure will continue as normal with cannulation of the vein in the standard fashion however those in the study group will have a second cannula placed in you groin to allow passage of the ultrasound into the pelvic veins. Notes will be taken during your procedure to document your procedural findings.
- After the procedure your data will be stored in University Hospital Galway where it will be stored securely within the Department of Vascular Surgery.
- When 60 participants have been recruited the study will be stopped and the information gathered will be analysed to assess whether assessment of the iliac veins was of any benefit to patients undergoing the procedure.
- Data will be anonymous throughout and not traceable back to individuals.
- Data will be stored within the Department for a period of 5 years for research only purposes.
- This information may in turn be published in medical journals in order to guide research and practice in this area.
- You will be followed up as standard in the outpatients department. Further appointments will be made at 6 and 12 weeks/ 6 and 12 months and annually thereafter.
- Should you have queries or concerns at any point one of the investigators will be available for contact.

**Potential Harms:**

- During the study the participant may have an additional cannula placed in the common femoral vein (groin). Furthermore, they may have an intravascular ultrasound probe placed into the pelvic veins and undergo stenting of any norrowed segments. This is a commonly performed procedure and has a very low risk of any side effects. Potential harms are listed below. There are known harms associated with venous surgery and additional harms associated with the extra procedure in the study group. It is important to note that there may be harms that we are as of yet unaware. If any contra-indications exist participation will not be possible.

**Harms and potential complications:**

- Pain
- Bleeding
- Need for blood transfusion
- Infection
- Allergic reaction to intraoperative medications
- Deep vein thrombosis
- Pulmonary embolus
- Nerve injury
- Recurrence of symptoms/venous incompetence
- Conversion to open surgery including iliac cut-down
- Iliac vein damage/occlusion
- Failure of ulcer healing
- Myocardial infarction
- Respiratory compromise
- Skin burns
- Phlebitis
- Contrast induced nephropathy

**Potential Benefits:**

- The most significant benefit to participants resulting from this study is a potentially improved chance of ulcer healing and a reduced chance of future recurrence after the procedure. This could potentially reduce the increased risk of complication related to chronic ulceration.

**Alternatives to participation:**

- If you choose not to partake in this study your procedure will proceed in the standard fashion. Your decision will result in no change to the planned procedure, its indications or its timing.

**Confidentiality:**

- We will respect your privacy. No information about who you are will be given to anyone or be published without your permission.
- The data produced from this study will be stored in a secure, locked location. Only members of the research team will have access to the data. This could include external research team members. Following completion of the research study, the data will be kept as long as required and then destroyed as required by the local ethics policy. Published study results will not reveal your identity.
- The results of the tests we describe in this form will be used only for this study

**Participation:**

- It is your choice to take part in this study. You can stop at any time. The care you get will not be affected in any way by whether you take part in this study.
- If you become ill or are harmed because of study participation, we will treat you.

**Conflict of Interest:**

- No research team member, has a conflict of interest to declare

**Consent**:

- By signing this form, I agree that:

1) You have explained this study to me. You have answered all my questions.

2) You have explained the possible harms and benefits (if any) of this study.

3) I know what I could do instead of taking part in this study. I understand that I have the right not to take part in the study and the right to stop at any time. My decision about taking part in the study will not affect my health care

4) I am free now, and in the future, to ask questions about the study.

5) I have been told that my medical records will be kept private except as described to me.

6) I understand that no information about who I am will be given to anyone

7) I have read and understood the pages of this consent form. I agree, or consent, to take part in this study.

8) I understand that my data may be anonymously shared with other researchers involved in this study

9) I understand if I withdraw from the trial I will be asked to state whether or not to withdraw my data (to that point) from the trial

_________________________________

Printed Name Subject’s signature & date

_________ _________________________________

Printed Name of person who explained consent Signature & date

_______________________________________ __________________________________

If you have any questions about this study, please call _________________at__________________

**Retention of signed research consent forms;**

The research consent form is a permanent part of the health record.
